# Supplementary material for: Genome-Resolved Co-Infection by Aeromonas veronii and Shewanella sp. in Koi Carp: A Zoonotic Risk for Aquarists
Source: Microorganisms. 2025 Dec 23;14(1):36. doi: 10.3390/microorganisms14010036 (PMC12844100; doi:10.3390/microorganisms14010036)
Supplement: Supplementary file 1 [file microorganisms-14-00036-s001.zip › microorganisms-4007701-supplementary.pdf]

Table S1. Genomes included in the BV-BRC phylogenomic analysis of the koi-derived isolate *Shewanella* sp. Koi-1.

Species	Strain	Host Group	Country/Region	Accession Number	Source
Shewanella xiamenensis	111D	Human	France	MWWO00000000	Stool
Shewanella xiamenensis	ZJ282	NR	China	JBHFGS000000000	River
Shewanella sp.	Sh95	Human	Argentina	LGYY00000000	Ocular Secretion
Shewanella xiamenensis	HD6452	NR	China	CP091834	Hospital Wastewater
Shewanella sp.	ZOR0012	Fish	USA	JTLE00000000	Intestinal Tract
Shewanella xiamenensis	ZYW1	Fish	Taiwan	LVDL00000000	NR
Shewanella xiamenensis	160P	Fish	Turkiye	JAZDQS000000000	NR
Shewanella xiamenensis	28.1.37	Fish	USA	JANLGK000000000	Skin Mucus
Shewanella xiamenensis	NF-3	Environment	USA	JAOTLY000000000	Zebrafish tank water
Shewanella seohaensis	KCTC 23556	Environment	China	CP104900	Sediment
Shewanella seohaensis	CCUG60900	Environment	South Korea	JAKILE000000000	Tidal Flat Sediments
Shewanella sp.	BC20	Fish	USA	NHRI00000000	Intestines
Shewanella seohaensis	SH314	Environment	China	JBHFGJ000000000	River
Shewanella sp.	Koi 1	Fish	Turkiye	JBREJM000000000.1	Head Kidney
Shewanella seohaensis	AH575	Environment	China	JBHFGO000000000	River

Shewanella sp.	Sh95	Human	Argentina	LGYY00000000	Ocular Secretion
Shewanella mangrovisoli	YQ_8	Fish	China	JBICJP000000000	Intestines
Shewanella sp.	MR-4	Environment	Black Sea	CP000446	Black Sea
Shewanella baltica	SF1039	Environment (Food origin)	France	OX461117	Smoked Salmon
Shewanella baltica	11FHM2	Fish	Norway	CP051529	Intestines
Shewanella baltica	Sheb_23	Marine Invertebrate	Egypt	AP031461	Squid Ink Sack
Shewanella sp.	SACH	Environment	Antarctica	MPDG00000000	Soil
Shewanella sp.	PP-Sp27a-2	Fish	Baltic Sea	JARLUH000000000	Skin
Shewanella baltica	CW2	Fish	USA	CP028355	Intestines
Shewanella sp.	RGIG9240	Deer	China	JAGBMC000000000	Gastrointestinal Tract
Shewanella baltica	M1	Environment	Poland	LWED00000000	Brackish Water
Shewanella sp.	N2AIL	Fish	India	JALBYT000000000	Gastrointestinal Tract
Shewanella oncorhynchi	S-1	Fish	Turkiye	JABAEB000000000	Eye
Shewanella putrefaciens	SRR6727992_bin.1_metaWRAP_v1.3_MAG	Fish	USA	CAMSMN000000000	Fish Metagenome
Shewanella morhuae	CW7	Fish	USA	PYSG00000000	Intestines
Shewanella putrefaciens	XY07	Fish	China	CP070865	NR
Shewanella putrefaciens	ATCC 8071	Human	USA	CP066370	Water
Shewanella putrefaciens	CGMCC-1.6515	Human	China	CP066369	Water

Shewanella sp.	Glo_24	Environment	Sweden	PIWH00000000	Hypoxic Fjord Sediment
Shewanella sp.	10N.286.48.B5	Environment	USA	MCVG00000000	Filtered Seawater
Shewanella sp.	H8	Environment	Antarctica	CP157213	Red Algae
Shewanella psychromarinicola	R106	Environment	Antarctica	RKKB00000000	Deep Sea Sediments
Shewanella sp.	Alg231_23	Environment	Portugal	FKJK00000000	Marine Sponge
Shewanella insulae	M13-18293	Marine Invertebrate	Australia	JAKEVE000000000	Pacific Oyster
Shewanella schlegeliana	JCM 11561	Fish	Japan	JAESVD000000000	Intestines
Shewanella sp.	yb_14	Fish	China	JBICJX000000000	Intestines
Shewanella algae	HIDE	Human	Taiwan	CP032664	Blood
Shewanella algae	MN01	Environment	USA	LIRM00000000	Salt Marsh Sediment
Shewanella algae	HUD-H4	Human	Sweden	JADZGR000000000	Wound
Shewanella algae	CCUG-56496	Human	Sweden	JADZHN000000000	Trachea
Shewanella algae	590722	Human	Spain	JADZHH000000000	Ear
Shewanella algae	254-1	Human	Spain	JAKILW000000000	Bile
Shewanella algae	159418	Human	Spain	JADZHI000000000	Ulcer
Shewanella algae	CCUG-72678	Human	Sweden	JADZHK000000000	Crushwound
Shewanella algae	B2215466	Human	Germany	CP110338	Blood
Shewanella algae	S12	Reptile	China	JBDIND000000000	Wound
Shewanella algae	HUD-G3	Human	Sweden	JADZGS000000000	Blood
Shewanella algae	SF7	Human	Spain	JADZGP000000000	Urinary Catheter Exudate
Shewanella algae	FFTB-27618	Human	Tanzania	CAXORA000000000	Wound
Shewanella algae	18064-CSB-B-B	Poultry	Tanzania	CP047422	Stool

Shewanella indica	INSAq347	Marine Invertebrate	Portugal	JAKCOQ000000000	Mussels
Shewanella indica	SYCDC19SW03	Human	China	JAVMLA000000000	Stool
Shewanella amazonensis	SB2B	Environment	Brazil	CP000507	Mud
Shewanella sp.	SNU WT1	Fish	South Korea	CP041151	Kidney
Shewanella sp.	NFH-SH190041	Fish	Japan	AP026070	Muscle

Table S2. Genomes included in the BV-BRC phylogenomic analysis of the koi-derived isolate *Aeromonas veronii* Koi-2.

Species	Strain	Host Group	Country/Region	Accession Number	Source
Aeromonas veronii	AvePhAetol1	Fish	Greece	JBLUPD000000000	Kidney
Aeromonas veronii	50A	Fish	Turkiye	NPKD00000000	Kidney
Aeromonas veronii	NS22	Fish	Greece	NQMC00000000	Kidney
Aeromonas veronii	CECT7059	Environment	Spain	NKWM00000000	Drinking Water
Aeromonas veronii	Hm21	Invertebrate	Turkiye	CP059397	Digestive Tract
Aeromonas veronii	UBA1835	Fish	Spain	DDJB00000000	Epidermal Mucus
Aeromonas veronii	AK247	Human	USA	NKXD00000000	Forehead Abscess
Aeromonas veronii	AK236	Environment	France	NKXF00000000	Lake Water
Aeromonas veronii	AS1	Human	Australia	CP114182	Intestinal Biopsy
Aeromonas veronii	C7_8	Environment	South Africa	JANTID000000000	Corn Plant Root
Aeromonas veronii	FES165	Swine	Italy	JAULJM000000000	Liver
Aeromonas veronii	ERR1305902-bin.15	Human	Denmark	CAJKQS000000000	Intestines
Aeromonas veronii	17.1.12	Fish	USA	JAMXHX000000000	Skin Mucus
Aeromonas veronii	A17	Fish	China	JBLNHM000000000	NR
Aeromonas veronii	FS120001	Fish	China	CP139189	Spleen
Aeromonas veronii	ALP17-18	Fish	USA	JAOTPU000000000	NR
Aeromonas veronii	Koi-2	Fish	Turkiye	JBREJN000000000	Heart
Aeromonas veronii	TCO21	Fish	USA	NKVZ00000000	Intestines
Aeromonas veronii	SD/21-14	Environment	Norway	JAJVCX000000000	Fresh water
Aeromonas veronii	VA60	Fish	Vietnam	AP027937	Intestines
Aeromonas veronii	GT1	Fish	Brazil	JAACNE000000000	Kidney
Aeromonas veronii	A03	Marine Invertebrate	China	JBLNHQ000000000	Pacific White Shrimp

Aeromonas veronii bv. sobria	BC88	Human	Australia	CAAKNH000000000	Dysentery patient
Aeromonas veronii	AGM2	Fish	Bangladesh	CP144910	Lesion
Aeromonas veronii	A16	Marine Invertebrate	China	JBLNHN000000000	Pacific White Shrimp
Aeromonas veronii	MS 17-88	Fish	USA	RAWX00000000	NR
Aeromonas veronii	CCSB46	Fish	Thailand	JBJOUJ000000000	Tissue
Aeromonas veronii	A20-12	Human	Australia	JAIEYF000000000	Stool
Aeromonas veronii	D	Environment	South Africa	VZQA00000000	Water
Aeromonas veronii	ZfB1	Fish	China	CP034967	NR
Aeromonas veronii bv. sobria	A4	Environment	Kenya	JBHFVN000000000	Fish Pond
Aeromonas veronii	0.14	Fish	Spain	JANLFC000000000	NR
Aeromonas veronii	FUJ01204	Human	Japan	JAPEGY000000000	Blood
Aeromonas veronii	DFR01	Fish	Philippines	JBEHWV000000000	NR
Aeromonas veronii	AK227	Environment	France	NKXG00000000	Wastewater Treatment Plant
Aeromonas veronii	C02	Environment	Spain	JBELAX000000000	Wastewater
Aeromonas veronii	AV066	Fish	China	CP126578	Liver, Spleen and Kidney
Aeromonas veronii	AV040	Fish	China	CP095841	Liver, Spleen and Kidney
Aeromonas veronii	C4	Fish	China	CP110364	NR
Aeromonas veronii	ML09-123	Fish	USA	PPUW00000000	NR
Aeromonas veronii	PLRT09	Fish	Thailand	JBJOUL000000000	Tissue
Aeromonas veronii	b1-3	Fish	China	CP091140	Intestines
Aeromonas veronii	CAPA018	Fish	Peru	JAPJZB000000000	Kidney and Liver

Table S3. Virulence and virulence-associated genes of *Shewanella* sp. Koi-1 isolate.

Strain	Virulence Mechanism	Virulence Gene(s)
***Shewanella* sp. Koi-1**	Adherence	*flgA, flgB, flgC, flgD, flgE, flgF, flgG, flgH, flgI, flgJ, flgM, flgN, flgO, flhA, flhB, flhG, flhF, fliP, fliR, fliI, fliH, fliA, fliE, , fliF, fliG, fliJ, fliK, fliL, fliM, fliN, fliO, motA, motB, pomA, pilB, pilM, pilN, pilQ, pilP, pilT/pilU, pilV, pilW, pilY/pilC, pilZ, mshF, mshI, mshJ, mshK, mshL, mshP, csgB, csgD, csgE, csgF, csgG*
	Chemotaxis and motility	*cheB, cheR, cheV, cheW, cheY cheZ*
	Iron uptake	*hemA, hemB, hemC, hemD, hemE, hemF, hemG, hemH, hemN, feoA, feoB, ccmA, ccmB, ccmC, ccmD, ccmE, ccmF, ccmG, ccmH, ccmI,*
	Quorum sensing	*luxS*
	Secretion system	*gspC, gspD, gspE, gspF, gspG, gspH, gspI, gspJ, gspK, gspL*
	Toxin	*cysC*
	Anaerobic respiration	*napA, napB, narQ*
	Efflux pump	*acrA, acrB, tolC*
	Endotoxin	*kdsA, lpxA, lpxB, lpxC, lpxD, lpxK, lpxL/lpxP, lpxM, msbA, rfaE, waaA*
	Immune evasion	*galE, galU, glmM, pgi, ugd, gcfC, wzi, fabA, fabB, fabD, fabF, fabG, fabH, fabV, fabR, fabZ, eptA, gmhA, arnT,*
	Nutritional virulence	*cydC, cydD, cysE, cysK, cysM, gshA, gshB, birA, bioA, bioB, bioC, bioD, bioF, accB*
	Protease	*zmp1 (Zn^{++} metallophrotease)*
	Serum resistance and immune evastion	*lptB, lptC, lptD, lptE, lptF, lptG*
	Stress adaptation	*katB, katG*

Table S4. Virulence and virulence-associated genes of *Aeromonas veronii* Koi-2 isolate obtained from the VFDB database.

Strain	Virulence Mechanism	Virulence Gene(s)
***Aeromonas veronii* Koi-2**	Adherence	*flgC, flgE, flgI, flgJ, fliF, fliG, fliP, lafB, lafC, lafF, lafK, lafS, lafT, lafU, lafX, lfgA, lfgB, lfgF, lfgG, lfgH, lfgK, lfgL, lfgM, lfgN, lfhA, lfhB, lfiE, lfiH, lfiI, lfiJ, lfiM, lfiN, lfiQ, lfiR, maf-5, mshA, mshB, mshC, mshD, mshE, mshF, mshG, mshI1, mshI, mshJ, mshK, mshL, mshM, mshN, mshO, mshP, cheA-2, cheB-2, cheR-3, cheV, cheW, cheY, cheZ, flaA, flaB, flaG, flaH, flaJ, flgA, flgB, flgC, flgD, flgE, flgF, flgG, flgH, flgI, flgJ, flgK, flgL, flgM, flgN, flhA, flhB, flhF, flhG, fliA, fliE, fliF, fliG, fliH, fliI, fliJ, fliK, fliL, fliM, fliN, fliO, fliP, fliQ, fliR, flmD, flmH, flrA, flrB, flrC, maf-1, maf-2, motX, motY, nueA, nueB, pomA2, pomA, pomB2, pomB, tapB, tapC, tapD, tapF, tapM, tapN, tapO, tapP, tapQ, tapT, tapU, tapV, tapW, tapY1, tppA, tppB, tppC, tppE, tppF*
	Secretion System	*exeA, exeB, exeC, exeD, exeE, exeF, exeG, exeH, exeI, exeJ, exeK, exeL, exeM, exeN, acr1, acr2, acrG, acrH, acrR, acrV, aexT, aopB, aopD, aopN, ascB, ascC, ascD, ascE, ascF, ascG, ascH, ascI, ascJ, ascK, ascL, ascN, ascO, ascQ, ascR, ascS, ascT, ascU, ascV, ascX, ascY, exsA, exsB, exsC, exsD, exsE, sycX, ppkA*
	Toxin	*hlyA, hly-III, th (thermostable hemolysin)*
	Fimbrial Adherence Determinants	*csgG*
	Immune Evasion	*wzi (Acinetobacter)*
	Iron Uptake	*entA, entB, entE,*

Table S5. Antimicrobial resistance (AMR) genes, proposed resistance mechanisms, and associated drug classes identified in *Shewanella* sp. Koi-1 and *Aeromonas veronii* Koi-2 isolates.

Strain	AMR Gene(s)	Proposed AMR Mechanism	Drug Class
***Shewanella* sp. Koi 1**	*OXA-436*	antibiotic inactivation	carbapenem, penicillin beta-lactam
	rsmA	antibiotic efflux	fluoroquinolone antibiotic, diaminopyrimidine antibiotic, phenicol antibiotic
	Escherichia coli EF-Tu mutants conferring resistance to Pulvomycin	antibiotic target alteration	elfamycin antibiotic
***Aeromonas veronii* Koi 2**	*sul1*	antibiotic target replacement	sulfonamide antibiotic
	qacEdelta1	antibiotic efflux	disinfecting agents and antiseptics
	cphA3	antibiotic inactivation	carbapenem
	rsmA	antibiotic efflux	fluoroquinolone antibiotic, diaminopyrimidine antibiotic, phenicol antibiotic
	tet(A)	antibiotic efflux	tetracycline antibiotic
	aadA3	antibiotic inactivation	aminoglycoside antibiotic
	OXA-1157	antibiotic inactivation	penicillin beta-lactam
	Escherichia coli EF-Tu mutants conferring resistance to Pulvomycin	antibiotic target alteration	elfamycin antibiotic

Table S6. Predicted secondary metabolite biosynthetic gene clusters identified in the *Shewanella* sp. Koi-1 genome using antiSMASH.

Region	Type	Genomic Coordinates (From-To)	Most Similar Known Cluster
Region 1.1.	RiPP-like	777,471 - 789,657	-
Region 1.2.	Terpene-precursor	1,454,410 - 1,475,291	-
Region 1.3.	hglE-KS, PUFA	1,520,406-1,576,693	eicosapentaenoic acid
Region 1.4.	RiPP-like	1,994,511 - 2,005,350	-
Region 1.5.	betalactone	2,813,306-2,844,722	corynecin III/corynecin 1/corynecinII
Region 1.6.	NI-siderophore	3,069,728-3,009,617	desferrioxamine E
Region 1.7.	RiPP-like	3,644,371 - 3,655,252	-
Region 1.8.	arylpolyene	4,429,744-4,473,617	APE Vf

Table S7. Predicted secondary metabolite biosynthetic gene clusters identified in the *Aeromonas veronii* Koi-2 genome using antiSMASH.

Region	Type	Genomic Coordinates (From-To)	Most Similar Known Cluster
Region 1.1.	terpene-precursor	831,520 - 852,410	-
Region 1.2.	RiPP-like	1,625,638 - 1,636,495	-
Region 1.3.	RiPP-like	2,643,307 - 2,655,067	-
Region 1.4.	NRP-metallophore,NRPS	3,004,449 - 3,053,053	chromobactin, NRPS:Type I
Region 1.5.	RiPP-like	3,322,067 - 3,332,915	-
Region 1.6.	arylpolyene	3,647,199 - 3,690,333	aryl polyenes
Region 1.7.	hserlactone	3,917,653 - 3,938,291	-

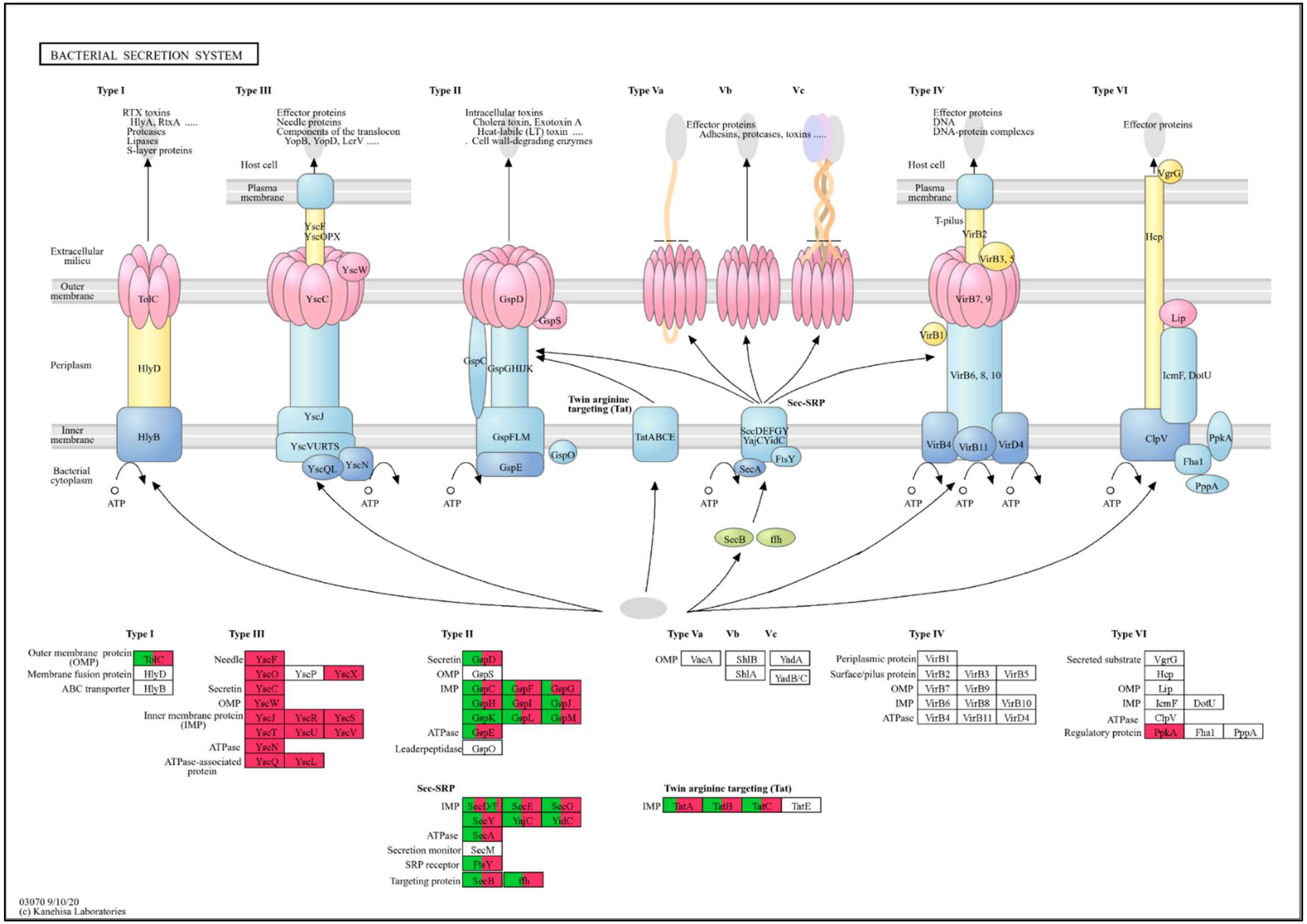

Figure S1. Bacterial secretion system pathways identified through KEGG Mapper analysis. Genes detected in *Shewanella* sp. Koi-1 are highlighted in green, while those in *Aeromonas veronii* Koi-2 are shown in red.

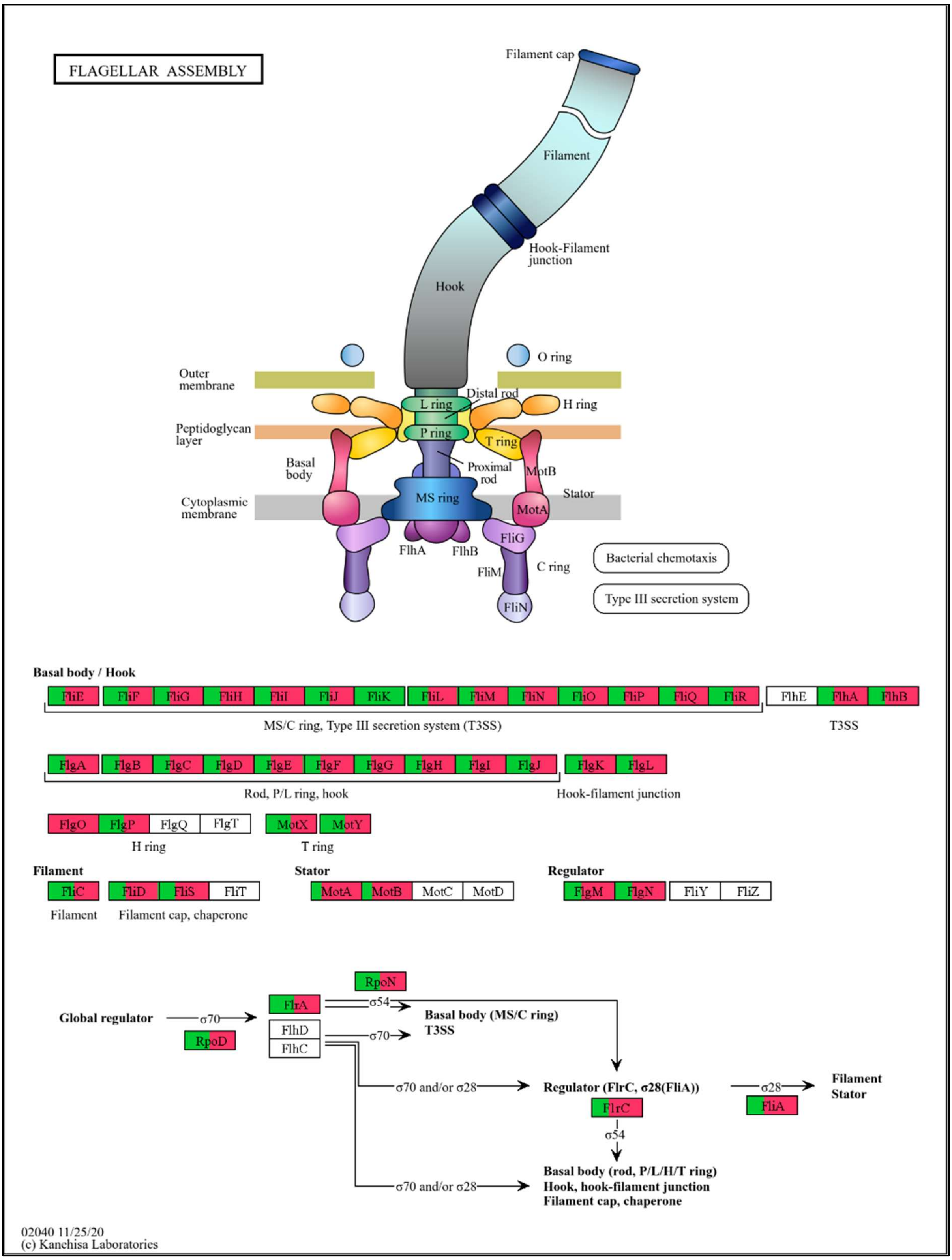

Figure S2. Flagellar assembly pathway reconstructed through KEGG Mapper showing genes involved in bacterial motility and host interaction. Genes detected in *Shewanella* sp. Koi-1 are shown in green, while those in *Aeromonas veronii* Koi-2 are shown in red.

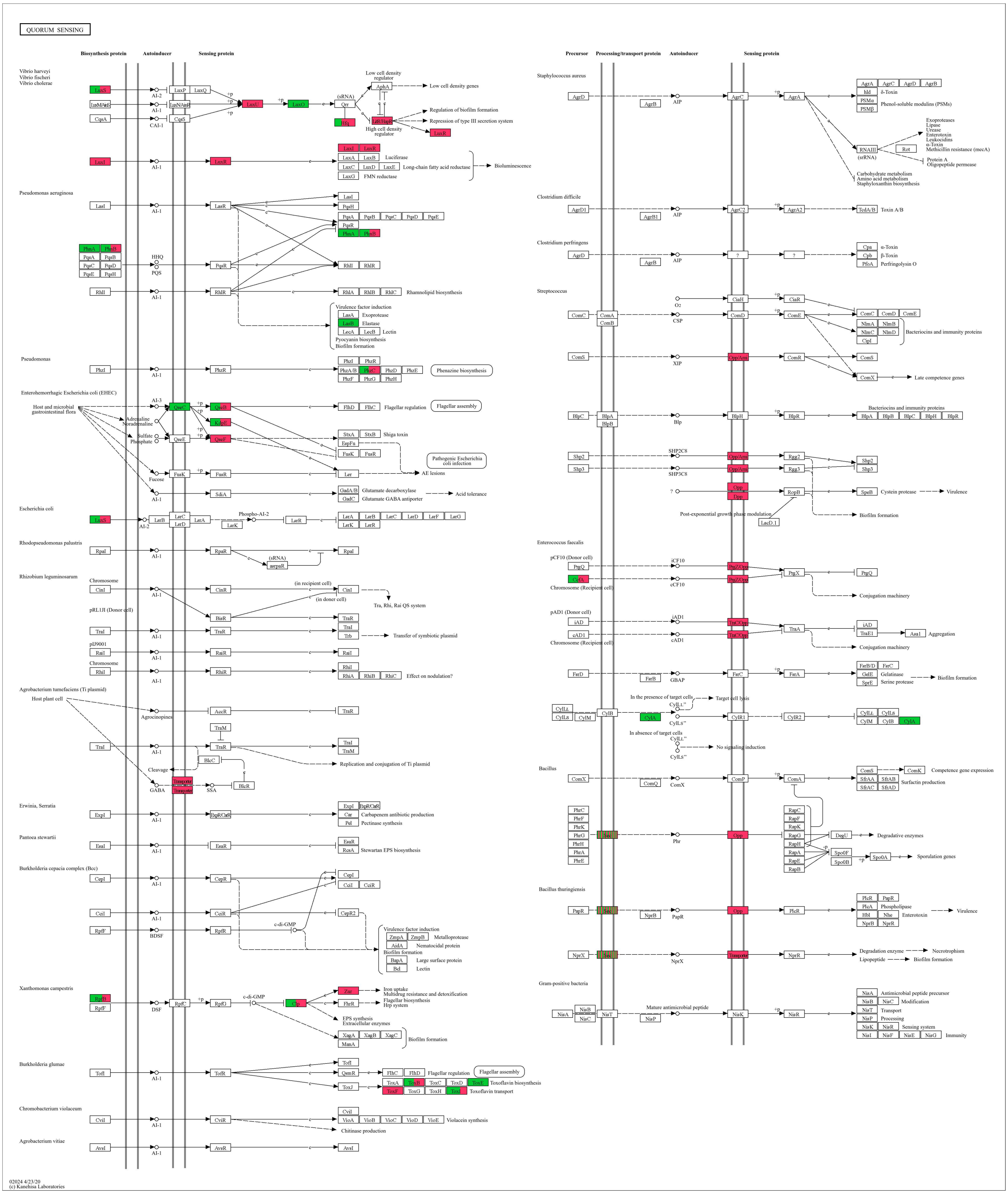

Figure S3. Quorum sensing pathway reconstructed through KEGG Mapper showing the distribution of genes involved in bacterial cell–cell communication. Genes detected in *Shewanella* sp. Koi-1 are highlighted in green, while those in *Aeromonas veronii* Koi-2 are shown in red.

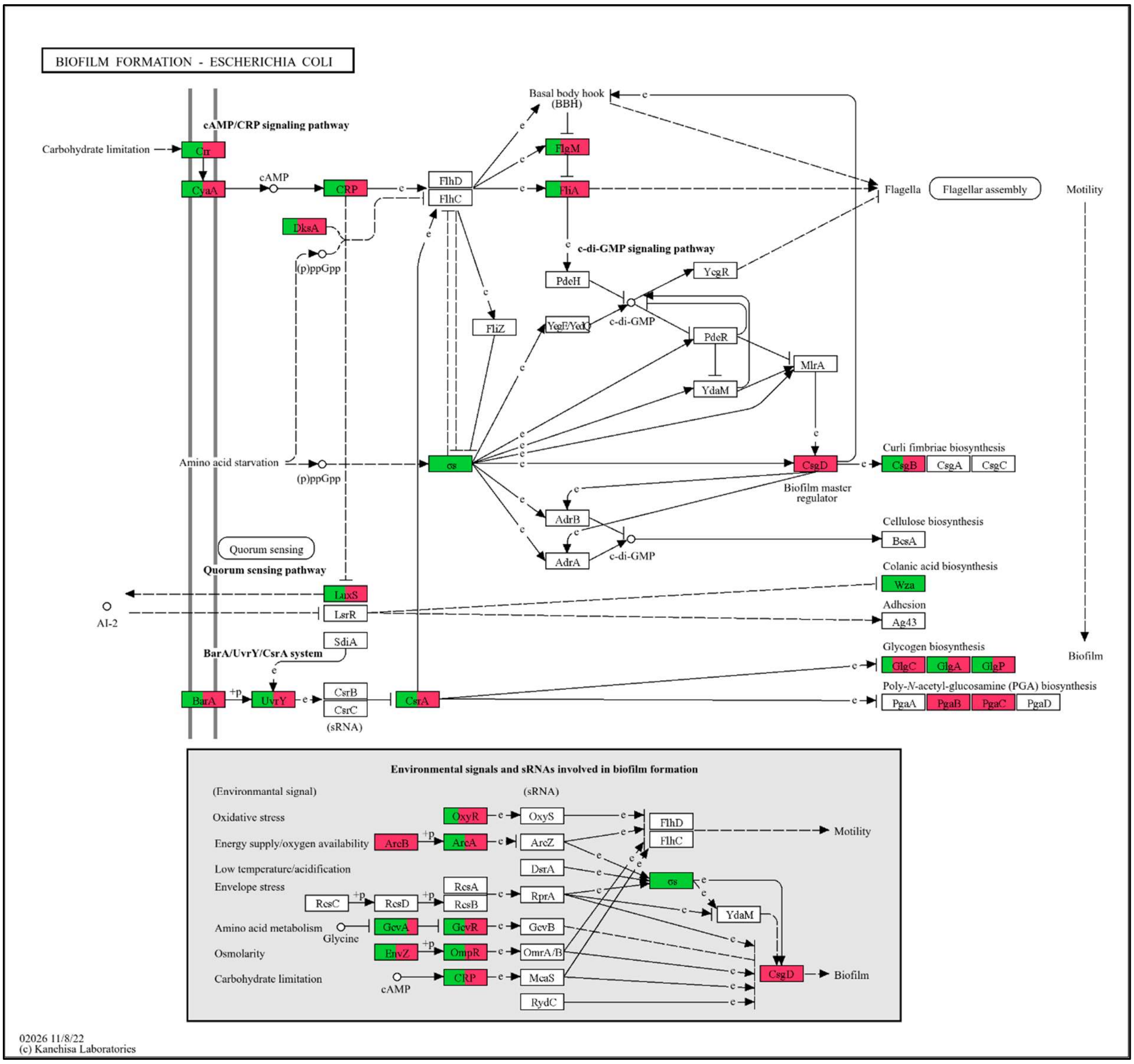

Figure S4. KEGG Mapper visualization of the biofilm formation pathway in *Escherichia coli* showing genes associated with adhesion, curli fiber production, and matrix regulation. Genes detected in *Shewanella* sp. Koi-1 are highlighted in green, and those in *Aeromonas veronii* Koi-2 are shown in red.

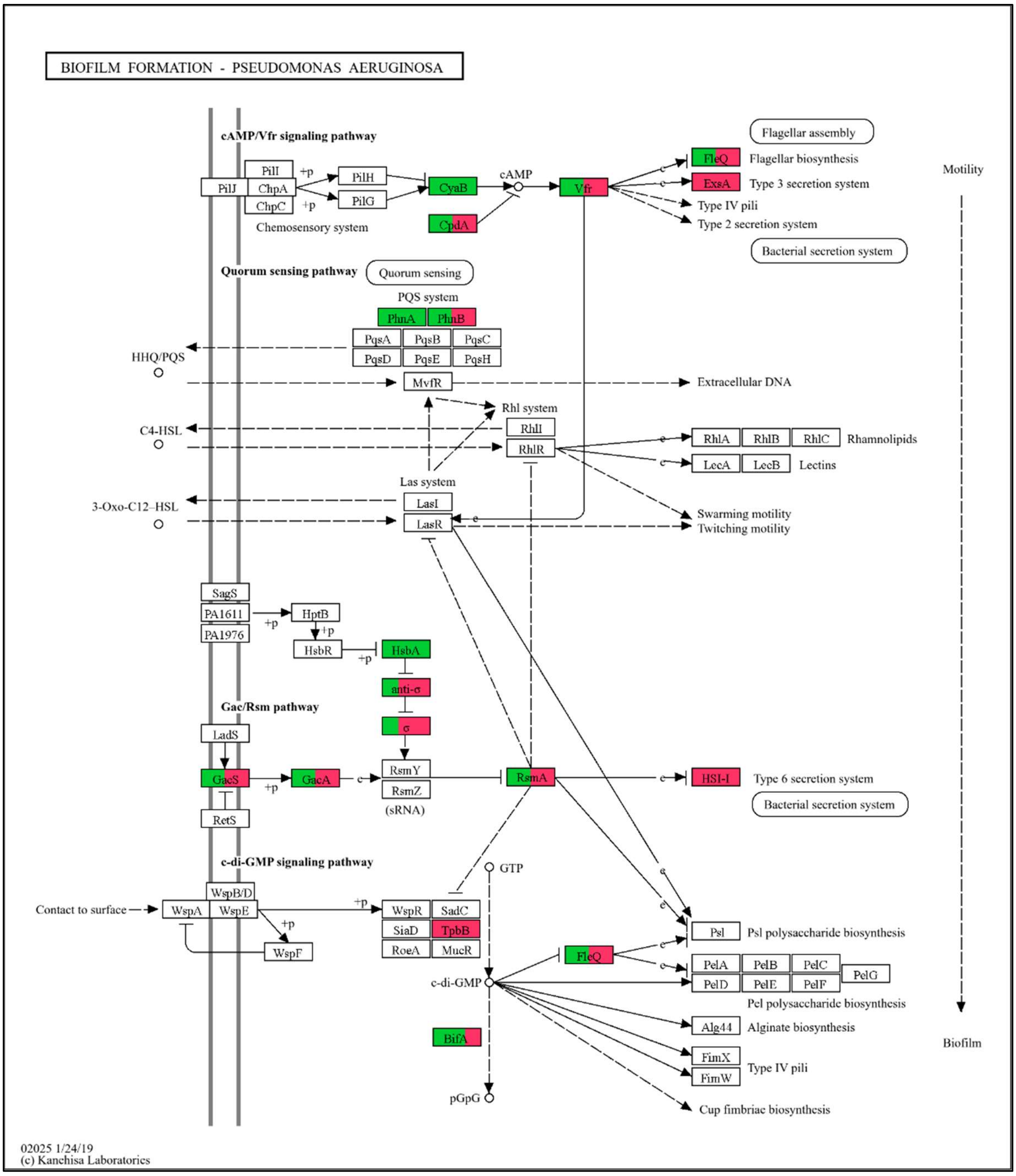

Figure S5. KEGG Mapper visualization of the biofilm formation pathway in *Pseudomonas aeruginosa* illustrating genes involved in EPS biosynthesis, quorum sensing, and motility regulation. Genes identified in *Shewanella* sp. Koi-1 are shown in green, and those in *Aeromonas veronii* Koi-2 are shown in red.

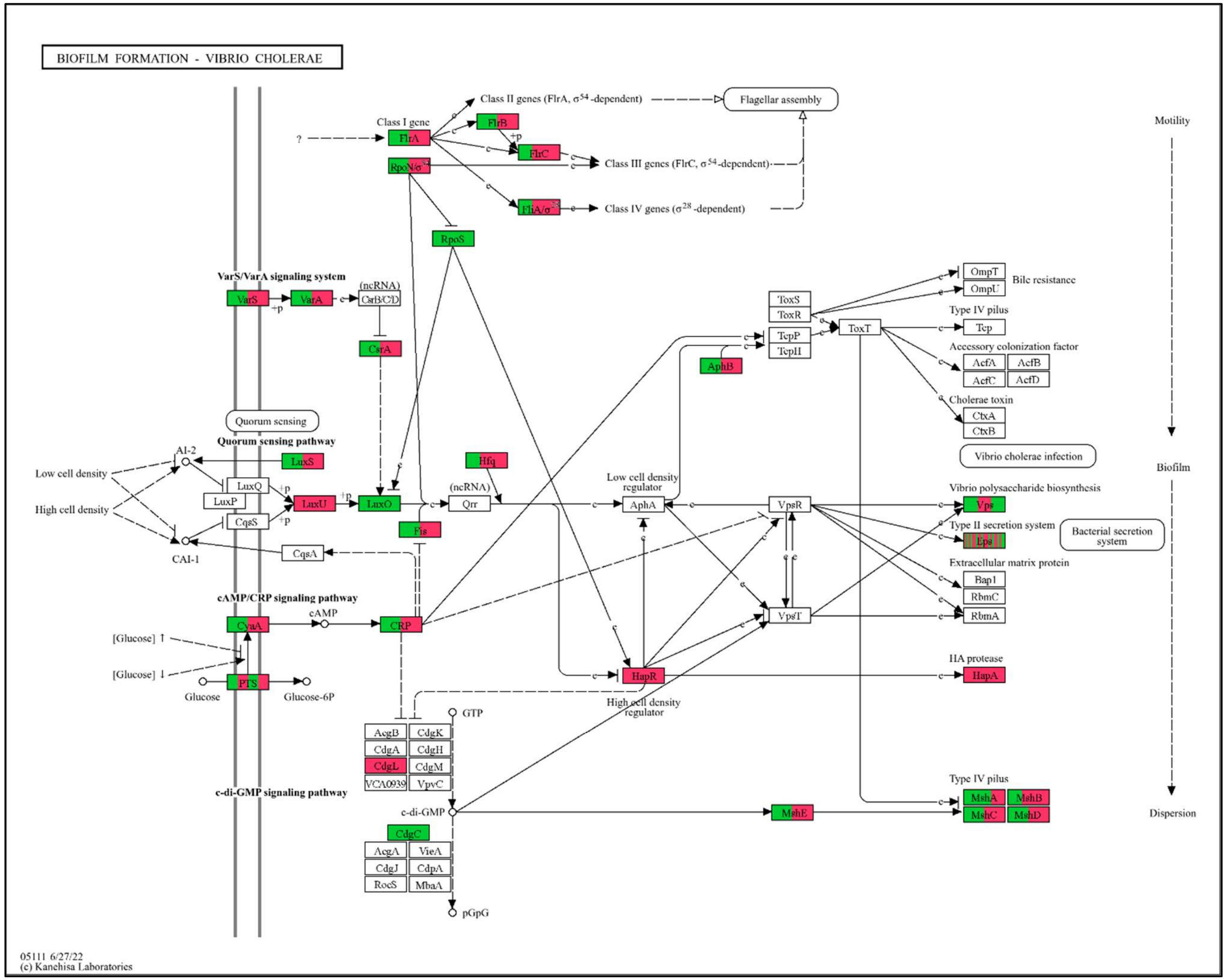

Figure S6. KEGG Mapper visualization of the biofilm formation pathway in *Vibrio cholerae* showing genes linked to biofilm matrix production, cyclic-di-GMP signaling, and cell-surface attachment. Genes detected in *Shewanella* sp. Koi-1 are shown in green, and those in *Aeromonas veronii* Koi-2 are shown in red.

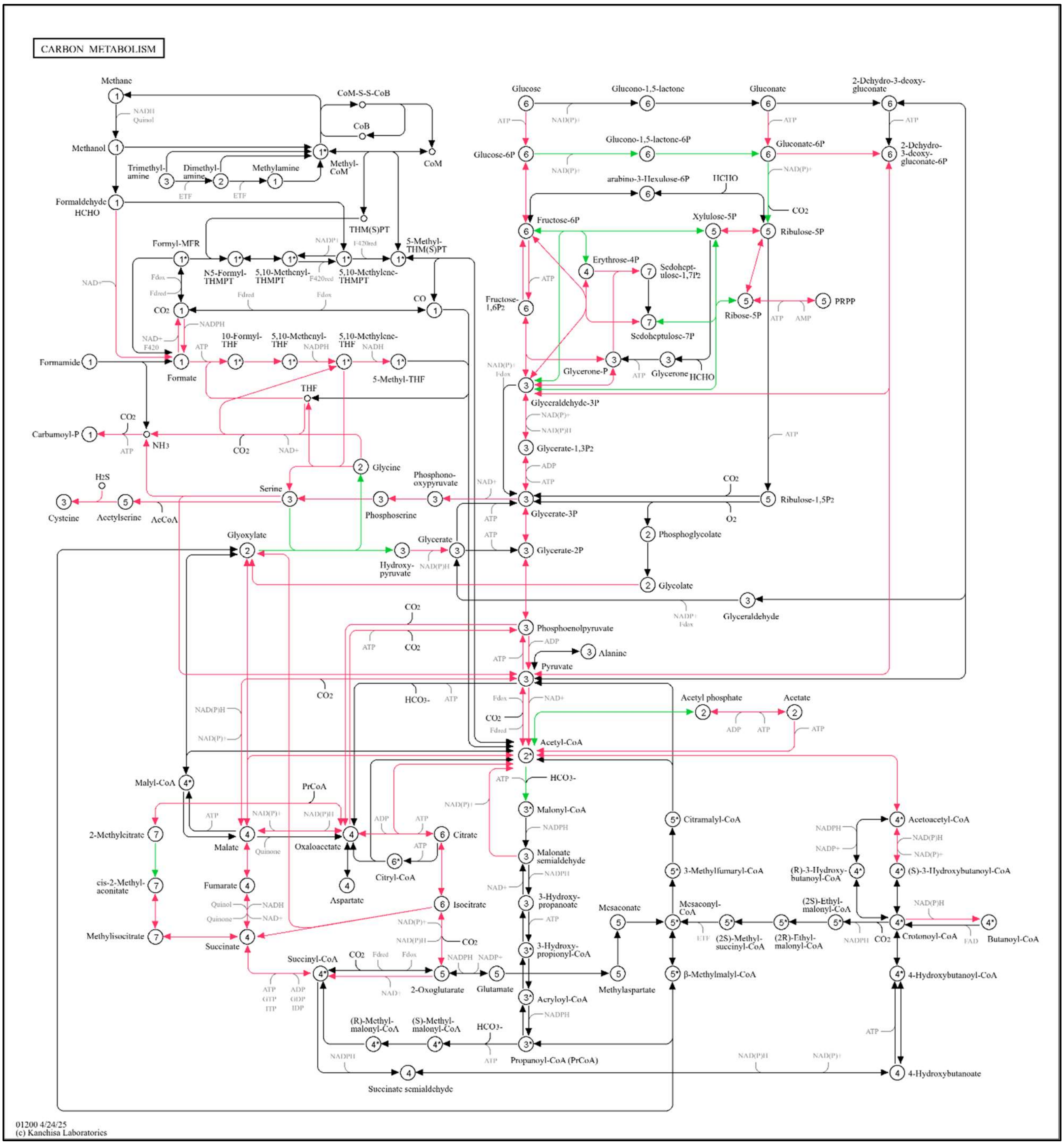

Figure S7. KEGG Mapper representation of the carbon metabolism pathway showing annotated enzymatic reactions associated with central carbon metabolism, energy production, and biosynthetic functions. Enzymatic steps predicted from the *Shewanella* sp. Koi-1 genome are shown in green, and those from *Aeromonas veronii* Koi-2 in red.

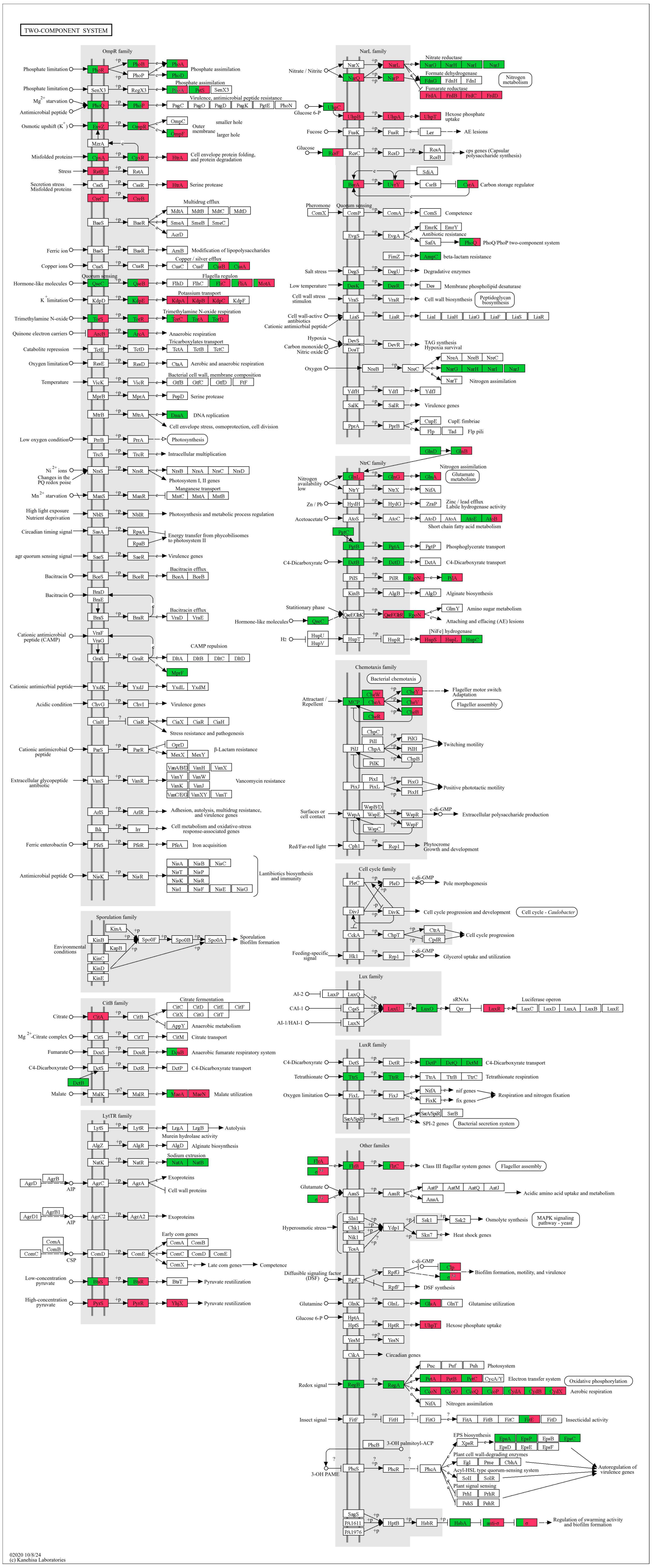

Figure S8. KEGG Mapper visualization of the two-component system pathway showing signal transduction genes involved in environmental sensing and regulatory responses. Genes detected in *Shewanella* sp. Koi-1 are highlighted in green, and those in *Aeromonas veronii* Koi-2 are shown in red.
